# Age-dependent increase of brain copper levels and expressions of copper regulatory proteins in the subventricular zone and choroid plexus

**DOI:** 10.3389/fnmol.2015.00022

**Published:** 2015-06-08

**Authors:** Sherleen Fu, Wendy Jiang, Wei Zheng

**Affiliations:** School of Health Sciences, Purdue UniversityWest Lafayette, IN, USA

**Keywords:** subventricular zone, choroid plexus, copper, age-dependent, neurogenesis

## Abstract

Our recent data suggest a high accumulation of copper (Cu) in the subventricular zone (SVZ) along the wall of brain ventricles. Anatomically, SVZ is in direct contact with cerebrospinal fluid (CSF), which is secreted by a neighboring tissue choroid plexus (CP). Changes in Cu regulatory gene expressions in the SVZ and CP as the function of aging may determine Cu levels in the CSF and SVZ. This study was designed to investigate the associations between age, Cu levels, and Cu regulatory genes in SVZ and plexus. The SVZ and CP were dissected from brains of 3-week, 10-week, or 9-month old male rats. Analyses by atomic absorption spectroscopy revealed that the SVZ of adult and old animals contained the highest Cu level compared with other tested brain regions. Significantly positive correlations between age and Cu levels in SVZ and plexus were observed; the SVZ Cu level of old animals was 7.5- and 5.8-fold higher than those of young and adult rats (*p* < 0.01), respectively. Quantitation by qPCR of the transcriptional expressions of Cu regulatory proteins showed that the SVZ expressed the highest level of Cu storage protein metallothioneins (MTs), while the CP expressed the high level of Cu transporter protein Ctr1. Noticeably, Cu levels in the SVZ were positively associated with type B slow proliferating cell marker *Gfap* (*p* < 0.05), but inversely associated with type A proliferating neuroblast marker *Dcx* (*p* < 0.05) and type C transit amplifying progenitor marker *Nestin* (*p* < 0.01). *Dmt1* had significant positive correlations with age and Cu levels in the plexus (*p* < 0.01). These findings suggest that Cu levels in all tested brain regions are increased as the function of age. The SVZ shows a different expression pattern of Cu-regulatory genes from the CP. The age-related increase of MTs and decrease of Ctr1 may contribute to the high Cu level in this neurogenesis active brain region.

## Introduction

Copper (Cu) is essential for numerous biological functions by serving as an indispensable cofactor for enzymes that widely involve in a number of biochemical reactions (Turski and Thiele, 2009; Uriu-Adams et al., 2010; Gambling et al., 2011; Zheng and Monnot, 2012). In the central nervous system, cuproenzymes (i.e., cytochrome C oxidase, superoxide dismutase, lysyl oxidase, ceruloplasmin, dopamine-β-monooxygenase, peptidylglycin α-amidating monoxygenase, and tyrosinase) participate in biological processes of energy metabolism, anti-oxidative defense, neurotransmitter and neuropeptide synthesis (Prohaska and Brokate, 2001; Takahashi et al., 2002; Skjørringe et al., 2012; Scheiber et al., 2014). Toxicologically, free Cu ions can interact readily with oxygen to initiate a cascade of reactions leading to the generation of highly damaging hydroxyl radicals. It is because of its essentiality to the cellular function and its cytotoxic nature in oxidative stress that Cu is strictly regulated in the body (Linder and Hazegh-Azam, 1996; Turnlund, 1998; Li and Zheng, 2005). Thus, disruption of the tightly regulated Cu homeostasis in the brain, either excess or deficient, can lead to severe neurological malfunction and neurodegeneration. A considerable amount of research has suggested that the pathogenesis of neurodegenerative disorders such as Parkinson’s disease (PD), Alzheimer’s disease (AD), familial amyotrophic lateral sclerosis (ALS), prion disease and the inherited disorders Menkes disease and Wilson’s disease, involve an imbalanced Cu homeostasis in the brain (Gaggelli et al., 2006; Matés et al., 2010; Zheng and Monnot, 2012). A recent human study compared the patterns of levels of biological metals in cerebrospinal fluid (CSF) among neurodegenerative diseases, and found that patients with PD, AD, or ALS had significantly higher Cu contents in the CSF than those of controls (Hozumi et al., 2011). Therefore, a stable Cu homeostasis must be carefully maintained to assure the normal brain function.

Cu from the blood circulation is transported into the brain via the blood-brain barrier (BBB) and blood-CSF barrier (BCB; Choi and Zheng, 2009; Zheng and Monnot, 2012; Fu et al., 2014). Previous studies from this laboratory have demonstrated that the BBB serves as the major route for the transport of Cu into the brain parenchyma, while the BCB mainly contributes to maintain the Cu homeostasis in the brain by exporting excess Cu from the CSF to the blood (Choi and Zheng, 2009; Zheng and Monnot, 2012; Fu et al., 2014). Once entering the brain, the Cu content and spatial distribution are uneven (Becker et al., 2005; Lech and Sadlik, 2007; Dobrowolska et al., 2008; Davies et al., 2013, 2014; Ramos et al., 2014), which also vary among different species (Waggoner et al., 2000; Olusola et al., 2004; Jackson et al., 2006), and change during the development, with age and in neurodegenerative conditions (Palm et al., 1990; Tarohda et al., 2004; Serpa et al., 2008; Wang et al., 2010; Ramos et al., 2014). Age-related increase in brain Cu content was observed in mouse (Wang et al., 2010), rat (Palm et al., 1990), and bovine (Zatta et al., 2008). Our recent published study using X-ray fluorescent (XRF) microscopy reveals an extraordinarily high Cu content in the subventricular zone (SVZ) along the wall of rat brain lateral ventricles, which appears to increase with age (Pushkar et al., 2013). More recently, our quantification studies by using atomic absorption spectrophotometry (AAS) further confirm that Cu concentrations in the SVZ are significantly higher than those in striatum (STR) and hippocampus (HP; Fu et al., 2015).

The SVZ, along with the subgranular zone (SGZ) in HP, serves as a source of neural stem/progenitor cells (NSPC) in the process of adult neurogenesis (Lledo et al., 2006; Ghashghaei et al., 2007). Actively differentiated NSPCs possess a unique ability to migrate from the SVZ origin, via the rostral migratory stream (RMS), to the olfactory bulb (OB; Lois et al., 1996; Curtis et al., 2007). On the migratory path, it is assumed that the cells may further differentiate in adjacent brain regions to provide renewed neurons so as to compensate the loss of neurons due to neurodegenerative injury (Lledo et al., 2006; Ghashghaei et al., 2007). Our recent studies have demonstrated that exposure to toxic metal manganese (Mn), which causes Parkinsonian disorders in humans (Crossgrove and Zheng, 2004; Racette et al., 2012), results in an elevated neurogenesis activity in the SVZ and RMS; the phenomenon appears to be associated with an increased expression of divalent metal transporter-1 (DMT1) in the SVZ (Fu et al., 2015).

Cellular Cu homeostasis is regulated by four major groups of Cu regulatory proteins, i.e., Cu uptake transporters such as Cu transporter 1 (CTR1) and DMT1, Cu efflux transporters such as Cu-transporting ATPases ATP7A and ATP7B, intracellular Cu chaperon proteins such as cytochrome c oxidase-17 (COX17), antioxidant 1 Cu chaperon (ATOX1), Cu chaperone for superoxide dismutase (CCS), and intracellular Cu binding proteins such as metallothioneins (MTs) for Cu storage (Scheiber et al., 2014). In mammalian brain, these Cu regulatory proteins are widely expressed with particular abundance in brain capillary endothelial cells and choroid plexus (CP) epithelial cells, the two major cell types constituting the BBB and BCB, respectively (Hidalgo et al., 2001; Kuo et al., 2006; Zheng and Monnot, 2012; Davies et al., 2013; Fu et al., 2014). Anatomically, the SVZ is a symmetric region located at the external wall of the both lateral ventricles. The SVZ has the direct contact with the CSF, which is secreted by the CP. It is therefore highly possible that the regulation of Cu transport across the BCB may influence the Cu accumulation in the SVZ. As an active neurogenesis region, the high Cu content in the SVZ may play a potential role in regulating the neurogenesis process. However, little knowledge is available on the expression patterns of Cu regulatory proteins in relationship to aging, as well as their contributions to the high Cu content in the SVZ.

The main purposes of this study were to: (1) compare the Cu distribution pattern in brain regions including SVZ, CP, HP, frontal cortex (FC), cerebellum (CB), and OB by using AAS; (2) assess the transcriptional expression levels of Cu regulatory proteins in the SVZ and CP tissues using quantitative real time RT-PCR (qPCR) and compare the expression patterns of Cu regulatory proteins between these two regions; and (3) investigate the associations between aging, Cu contents, and expressions of Cu regulatory proteins in the SVZ and CP tissues. The results of this study provide first hand evidence of Cu regulatory proteins in the SVZ and help establish a new connection between the SVZ and CP.

## Materials and Methods

### Materials

Chemical reagents were purchased from the following sources: cDNA synthesis kit and iTaq Universal SYBR Green Supermix from Biorad (Hercules, CA, USA), ultrapure nitric acid (HNO_3_) from Mallinckrodt (St. Louis, MO, USA), and other routine chemicals and reagents from Sigma (St. Louis, MO, USA). All reagents were of analytical grade, HPLC grade, or the best available pharmaceutical grade.

### Animals

Young (3 weeks), adult (10 weeks), and old (9 months) male Sprague-Dawley (SD) rats were purchased from Harlan Sprague Dawley Inc. (Indianapolis, IN, USA). Upon arrival, rats were housed in a temperature-controlled room under a 12-h light/12-h dark cycle and allowed to acclimate for 1 week prior to experimentation. They had free access to deionized water and pellet Purina semi-purified rat chow (Purinal Mills Test Diest, 5755C. Purina Mills, Richmond, Inc., USA). The study was conducted in compliance with standard animal use practices and approved by the Animal Care and Use Committee of Purdue University.

Rats were anesthetized with ketamine/xylaxine (75:10 mg/kg, 1 mg/kg i.p.). CSF samples, free of blood, were collected using 26G butterfly needle by inserting the needle between the protuberance and the spine of the atlas, and blood samples were obtained from the vena cava for serum separation. Rat brains were dissected to harvest the CP in lateral and third ventricles, SVZ, OB, STR, HP, FC, and CB for measurement of Cu levels using AAS. SVZ and plexus tissues were further used to determine the transcriptional expression levels of Cu binding proteins and cellular markers for SVZ neural stem cells using qPCR. Samples were freshly analyzed or stored at −80°C for further analyses.

### Determination of Copper Concentrations by AAS

Brain tissue and serum samples were digested with concentrated ultrapure HNO_3_ in a MARSXpress microwave-accelerated reaction system. Due to the small massive weights and volumes, as well as avoiding the over-dilution, SVZ, CP and CSF samples were digested overnight with 50–100 μL HNO_3_ in the oven at 55°C. An Agilent Technologies 200 Series SpectrAA with a GTA 120 graphite tube atomizer was used to quantify Cu concentrations. Digested samples were diluted by 5 or 10 times with 0.1% (v/v) HNO_3_ in order to keep the reading within the concentration range of the standard curve. Range of calibration standard for Cu was 0–25 μg/l. Detection limit for Cu was 0.9 ng/ml of the assay solution. Intra-day precision was 1.6% and the inter-day precision as 3.7% (Zheng et al., 1998, 1999, 2009).

### Quantitative Real Time RT PCR

Transcription levels of mRNA encoding Cu transporters (i.e., *Ctr1, Dmt1, Atp7a* and *Atp7b*), Cu chaperons (i.e., *Cox17, Ccs* and *Atox1*), Cu binding protein MTs (i.e., *Mt1a, Mt2a* and *Mt3*), and cellular markers for neuronal precursor cells (i.e., *Nestin, Dcx* and *Gfap*) were quantified using qPCR. Total RNA was isolated from rat SVZ and CP tissues by using TRIzol reagent following the manufacturer’s instructions. An aliquot of RNA (1 μg) was reverse-transcribed into cDNA using the BioRad iScript cDNA synthesis kit. The iTaq Universal SYBR Green Supermix was used for qPCR analyses. The amplification was run in the CFX Connect^TM^ Real-Time PCR detection system with an initial 3 min denaturation at 95°C, the amplification program was followed by 40 cycles of 30 s denaturation at 95°C, 10 s gradient from 55 to 65°C and 30 s extension at 72°C. A dissociation curve was used to verify that the majority of fluorescence detected could be attributed to the labeling of specific PCR products, and to verify the absence of primer dimers and sample contamination. Each qPCR reaction was run in triplicate. The relative mRNA expression ratios between groups were calculated using the delta-delta cycle time formulation. After confirming that the reference gene was not changed, the cycle time (Ct) values of interested genes were normalized with that of the reference gene in the same sample to obtain ΔΔCt values. The amplification efficiencies of target genes and the internal reference were examined by determining the variations of the cycle time with a series of control template dilutions.

The forward and reverse primers for target genes were designed using Primer Express 3.0 software and showed as follows:

**Table:** 

Category	Gene name	Primer sequence
Transporters	*Ctr1*	Forward	5′-TCG GCC TCA CAC TCC CAC GA-3′
		Reverse	5′-CGA AGC AGA CCC TCT CGG GC-3′
	*Dmt1*	Forward	5′-TCG CAG GCG GCA TCT TGG TC-3′
		Reverse	5′-TAC CGA GCG CCC ACA GTC CA-3′
	*Atp7a*	Forward	5′-CTT GTA GAG GAG GCA CAG AC-3′
		Reverse	5′-GGT AAC AAT GGA AAC CAA GA-3′
	*Atp7b*	Forward	5′-AAT CCA GGA CTG TCC GTT CTA A-3′
		Reverse	5′-CAC TTG CTC CTC TCT GAG GAT T-3′
Chaperons	*Cox17*	Forward	5′-CTG AGT TTT GGG AGC TTT GC-3′
		Reverse	5′-AGG GCT TCA GAG GCT TCT TC-3′
	*Ccs*	Forward	5′-TCA CAG GGA ATT CTG GGA AG-3′
		Reverse	5′-GGA GGC TCT GTT CAG AGG TG-3′
	*Atox1*	Forward	5′-CTC AAC AAA ACA GGA AAA GC-3′
		Reverse	5′-GAT CAA CAG TCT GCC TCT TC-3′
Binding Proteins	*Mt1a*	Forward	5′-GCC TTC TTG TCG CTT ACA CC-3′
		Reverse	5′-AGG AGC AGC AGC TCT TCT TG-3′
	*Mt2a*	Forward	5′-ACA GAT GGA TCC TGC TCC TG-3′
		Reverse	5′-GAG AAC CGG TCA GGG TTG TA-3′
	*Mt3*	Forward	5′-CCC TGC AGG ATG TGA GAA GT-3′
		Reverse	5′-TTT GCT GTG CAT GGG ATT TA-3′
Reference Gene	*Actb*	Forward	5′-AGC CAT GTA CGT AGC CAT CC-3′
		Reverse	5′-CTC TCA GCT GTG GTG GTG AA-3′

All primers were obtained from Integrated DNA Technologies (Coralville, IA, USA). Experimental conditions were optimized for annealing temperature, primer specificity and amplification efficiency.

### Statistical Analyses

All data are presented as mean ± SD. Statistical analyses of the differences among different age groups or different primary cells were carried out by one-way ANOVA with *post hoc* comparisons by the *Dunnett’s test* using IBM SPSS for Windows (version 22.0). Pearson correlation analysis was conducted to analyze the relationship among the test parameters. A Grubb’s test was used to screen outliers in AAS and qPCR data. In all cases, a probability level of p value equal to or less than 0.05 was considered as the criterion of significance.

## Results

### Brain Regional Copper Content

To determine whether the age affects the brain regional Cu contents, brain tissues of SVZ, plexus, OB, STR, HP, FC, CB, as well as CSF and serum were collected from three groups of rats with different ages for quantitation of Cu concentrations using AAS. By comparison between age groups, our results revealed that Cu levels of all selected brain regions increased as age progressed; Cu concentrations were significantly higher in the old age group than those in the young or adult groups (*p* < 0.05; Table 1). There were about 4–9 fold increases in brain tissue Cu levels in old animals compared with the young. The Cu content in the CSF of old rats was about 1.7-fold and 2.0-fold higher than those of the young and adult animals, respectively (*p* < 0.05; Table 1). Interestingly, the serum Cu level in the old age group was also found to be significantly higher, by 3.9-fold and 2.6-fold, than those of the young and adult groups, respectively (*p* < 0.01; Table 1). In addition, the adult rats had higher Cu levels in the STR (*p* < 0.01), CB (*p* < 0.05) and serum (*p* < 0.01) than those in the young rats (Table 1). These findings clearly establish a general age-dependent increase of Cu levels in different brain regions, CSF and serum.

**Table 1:** **Age-dependent increased brain regional copper levels**.

Samples	Cu concentration (μg/g tissue or μg/L)		
	Young rats	Adult rats	Old rats
SVZ	0.711 ± 0.052	0.928 ± 0.105	5.347 ± 0.563**^##^
CP	0.809 ± 0.256^a^	0.748 ± 0.182^aa^	3.352 ± 1.024**^##aa^
OB	0.554 ± 0.028^aabb^	0.735 ± 0.058^aa^	5.226 ± 0.513**^##bb^
STR	0.633 ± 0.062^bb^	0.832 ± 0.066**^ac^	2.880 ± 0.249**^##aacc^
HP	0.537 ± 0.035^aabbd^	0.633 ± 0.035^aabcdd^	3.149 ± 0.354**^##aacc^
FC	0.569 ± 0.039^aabbd^	0.585 ± 0.023^aabccdd^	2.227 ± 0.186**^##aabbccdee^
CB	0.518 ± 0.040^aabb^	0.569 ± 0.037*^aabbccdd^	2.417 ± 0.055**^##aabbccee^
CSF	14.29 ± 4.65	12.32 ± 5.94	24.32 ± 9.41*^#^
Serum	318.5 ± 25.87	476.6 ± 42.24**	1252.7 ± 109.3**^##^

Note: Data represents mean ± S.D., n = 8-12 (brain tissues), n = 5-7 (CSF) and n = 6-7 (serum). 1. Comparison among different age groups: *p < 0.05, **p < 0.01, as compared with young rats; ^#^p < 0.05, ^##^p < 0.01, as compared with adult rats. Comparison within the same age group: a: p < 0.05, aa: p < 0.01, as compared with SVZ; b: p < 0.05, bb: p < 0.01, as compared with CP; c: p < 0.05, cc: p < 0.01, as compared with OB; d: p < 0.05, dd: p < 0.01, as compared with STR; ee: p < 0.01, as compared with HP. SVZ, subventricular zone; CP, choroid plexus; OB, olfactory bulb; STR, striatum; HP, hippocampus; FC, frontal cortex; CB, cerebellum; CSF, cerebrospinal fluid.

Among young animals, the CP has the highest Cu content as compared with the other brain regions (*p* < 0.05), followed by SVZ, STR, FC, OB, and CB (Table 1). However, the highest Cu level was detected in the SVZ of adult and old rats, as compared with the other selected brain regions (Table 1). Animals in adult age showed higher Cu levels in the STR, CP, and OB, which were significantly higher than those in the HP, FC, and CB (*p* < 0.05; Table 1). Interestingly, the second highest Cu content in the old age group was observed in the OB, which was slightly lower than that in the SVZ but significantly higher than the level in the CP (*p* < 0.01; Table 1). These data indicate that both SVZ and CP accumulate a higher Cu than the other selected brain regions. Considering the adjacent anatomic locations of the SVZ and plexus, the higher Cu may imply the greater demand of both regions for Cu ions and/or for Cu regulation within brain parenchyma.

### Transcriptional Expression of Copper Regulatory Proteins in the SVZ and Choroid Plexus

A higher accumulation of Cu in a particular brain region could be due to a higher expression of Cu uptake transporters or intracellular binding proteins. The current literature lacked such information. Thus, as the first step to understand the Cu regulatory mechanism in the SVZ and the nearby CP, we determined the expression levels and identified the expression patterns of Cu regulatory proteins in the SVZ and plexus of rats with different ages. The mRNAs study included Cu transporters (i.e., *Ctr1, Dmt1, Atp7a* and *Atp7b*), Cu chaperons (i.e., *Cox17, Ccs* and *Atox1*) and Cu storage protein MTs (i.e., *Mt1a, Mt2a* and *Mt3*). After normalizing with the *Actb*, the ΔΔCt values were summarized in Table 2.

**Table 2:** **Transcriptional expression levels of copper regulatory proteins in SVZ and choroid plexus of young, adult and old rats**.

		SVZ (ΔΔ Ct value)			Choroid plexus (ΔΔ Ct value)		
Category	Gene	Young rats	Adult rats	Old rats	Young rats	Adult rats	Old rats
Transporters	*Ctr1*	0.0145 ± 0.0007	0.0105 ± 0.0009^aa^	0.0095 ± 0.0010^aa^	0.4364 ± 0.0817**	0.5291 ± 0.0568^aa##^	0.5904 ± 0.0705^aabΔΔ^
	*Dmt1*	0.0108 ± 0.0017	0.0095 ± 0.0028	0.0126 ± 0.0024^b^	0.0294 ± 0.0033**	0.0350 ± 0.0037^aa##^	0.0440 ± 0.0041^aabbΔΔ^
	*Atp7a*	0.0016 ± 0.0003	0.0016 ± 0.0003	0.0017 ± 0.0003	0.0152 ± 0.0040**	0.0131 ± 0.0017^a##^	0.0149 ± 0.0021^ΔΔ^
	*Atp7b*	0.0013 ± 0.0001	0.0009 ± 0.0003	0.0015 ± 0.0004^bb^	0.0010 ± 0.0004	0.0008 ± 0.0001	0.0009 ± 0.0002
Chaperones	*Cox17*	0.0547 ± 0.0109	0.0531 ± 0.0081	0.0663 ± 0.0297	0.1052 ± 0.0244**	0.1189 ± 0.0082^##^	0.1244 ± 0.0210^aΔΔ^
	*Atox1*	0.0024 ± 0.0024	0.0027 ± 0.0011	0.0027 ± 0.0013	0.0226 ± 0.0046**	0.0125 ± 0.0027^aa##^	0.0118 ± 0.0034^aabbΔΔ^
	*Ccs*	0.0207 ± 0.0055	0.0244 ± 0.0058	0.0323 ± 0.0125^aa^	0.0555 ± 0.0105**	0.0634 ± 0.0050^##^	0.0816 ± 0.0082^aabbΔΔ^
Binding Proteins	*Mt1a*	0.0630 ± 0.0125	0.0406 ± 0.0138^aa^	0.0985 ± 0.0282^aabb^	0.0560 ± 0.0131	0.0319 ± 0.0053^aa^	0.0483 ± 0.0126^bbΔΔ^
	*Mt2a*	0.2289 ± 0.0461	0.1288 ± 0.0515^aa^	0.2648 ± 0.1049^bb^	0.0247 ± 0.0159**	0.0119 ± 0.0032^##^	0.0189 ± 0.0133^ΔΔ^
	*Mt3*	0.2330 ± 0.0618	0.2970 ± 0.1008	0.8869 ± 0.3864^aabb^	0.1349 ± 0.0715**	0.0473 ± 0.0059^aa##^	0.1414 ± 0.0237^bbΔΔ^

Note: Data represents mean ± S.D., *n* = 6. SVZ, subventricular zone. Comparison between SVZ and choroid plexus with the same age: **p < 0.01, as compared with the SVZ of young rats; ^##^p < 0.01, as compared with the SVZ of adult rats; ΔΔ: p < 0.01, as compared with the SVZ of old rats. Comparison among different age groups within SVZ or choroid plexus tissues: a: p < 0.05, aa: p < 0.01, as compared with young rats; b: p < 0.05, bb: p < 0.01, as compared with adult rats.

Our qPCR ΔΔCt results demonstrated that all selected Cu regulatory proteins existed in both SVZ and CP of young, adult and old rats (Table 2). Noticeably, the pattern of transcriptional expression of these Cu regulatory proteins in the SVZ was quite different from that in the CP. The mRNA levels of Cu storage protein metallothionein (i.e., *Mt3, Mt2a* and *Mt1a*) were the highest in the SVZ tissues of all three ages, followed by Cu chaperons of *Cox17* and *Ccs*, and Cu transporters *Ctr1* and *Dmt1* (Table 2). In contrast, the CP of all age groups had the highest expression of Cu uptake transporter *Ctr1*, followed by *Mt3, Cox17, Ccs* and *Dmt1* (Table 2). When the comparison was made within the same tissue but different age groups, we observed significantly increased expressions of *Dmt1, Atp7b, Ccs*, and *Mts* (*Mt1a, Mt2a* and *Mt3*) in the SVZ of the old rats, while the expression of *Ctr1* showed a significant reduction in adult and old animals than the young animals (Table 2).

Among the age-dependent up-regulation, *Mt3* expression in the SVZ of old animals appeared to increase the most, about 3.8-fold (*p* < 0.01) and 3.0-fold (*p* < 0.01) increase, as compared with the young and adult age groups, respectively (Table 2). In the CP, however, the age-related mRNA expression appeared to be most abundant for *Ctr1* (*p* < 0.01), *Dmt1* (*p* < 0.01), Cu chaperons *Cox17* (*p* < 0.05) and *Ccs* (*p* < 0.01) when the values in the old animals were compared with those in the young animals (Table 2). Interestingly, significantly lower expressions of Cu chaperone *Atox1*, Cu binding proteins *Mt1a* and *Mt3* were observed in old animals, as compared with those in the young or adult groups (Table 2).

In comparison with the CP, the SVZ from all the ages showed significantly higher expressions of *Mt3* (*p* < 0.01) and *Mt2a* (*p* < 0.01) than those in the plexus, whereas the CP of all ages had more abundant expressions of Cu transporters (*Ctr1, Dmt1* and *Atp7a*) and Cu chaperons (*Cox 17, Atox1* and *Ccs*) than those in the SVZ (*p* < 0.01; Table 2). These findings demonstrate entirely different patterns of Cu regulatory proteins between the SVZ and CP. The high abundance of Cu binding proteins in the SVZ suggests that the SVZ may have the ability to store large amount of Cu ions in brain ventricular region, which underlies the high Cu content detected in this region (Table 1). Similarly, the high abundance of Cu transport proteins in the CP suggests that the BCB plays an important role in transporting Cu ions between the blood and CSF, which may influence the SVZ.

### Age-Depedent Expressions of Cellular Markers for Neural Stem Cells in SVZ

**Figure 1:**
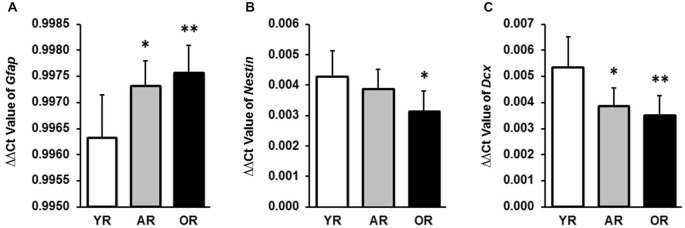
**Transcriptional expression levels of *Gfap, Nestin* and *Dcx* in subventricular zone (SVZ) tissues of young, adult and old rats by qPCR. (A)** mRNA expression levels of *Gfap* in SVZ tissues from all ages were expressed as the ΔΔCt value by normalizing with the *Actb*. **(B)** mRNA expression levels of *Nestin* in SVZ tissues were expressed as the ΔΔCt value by normalizing with the *Actb*. **(C)** mRNA expression levels of *Dcx* in SVZ tissues were expressed as the ΔΔCt value by normalizing with the *Actb*. The data are representative of triplicate experiments. Data represent mean ± SPRAGUE-DAWLEY (SD), *n* = 6; *p < 0.05, **p < 0.01, as compared with the YR. YR, AR, and OR are the abbreviations of young, adult and old rats.

The SVZ possesses four different types of cells: (i) type E ependymal cells; (ii) type A proliferating neuroblasts (Doublecortin (DCX) positive); (iii) type B slow proliferating progenitor cells (Nestin and glial fibrillary acidic protein (GFAP) positive); and (iv) type C transit amplifying progenitors (Nestin positive). In our preliminary study, by using qPCR we detected the expression of mRNAs encoding three NSPC markers (GFAP, Nestin and DCX) in the SVZ tissue; the results confirmed the validity of the dissection method used to collect the SVZ tissue from rat brain. The age effect on these NSPC markers was then investigated. Significant age-related expressions of these NSPC markers (*Gfap, Nestin* and *Dcx*) were found in the SVZ from three age groups (Figure 1). Specifically, *Gfap* transcriptional levels in the SVZ were significantly higher in the adult (*p* < 0.05) and old animals (*p* < 0.01) than that in the young rats (Figure 1A). A significant reduction of *Nestin* expression was observed in the old age group, as compared with the young animals (*p* < 0.05; Figure 1B). Both adult (*p* < 0.05) and old (*p* < 0.01) age groups showed a lower expression of *Dcx* than that of the young animals (Figure 1C).

### Correlations of Age-Dependent Copper Levels and Transcriptional Expressions of Copper Regulatory Proteins in SVZ and Choroid Plexus

We further extended our research to investigate the correlations among age, brain Cu content and Cu regulatory protein expressions in the SVZ (Table 3) and the CP (Table 4). Pearson correlation analyses revealed the following strong positive correlations in the SVZ: age and SVZ Cu content (Pearson *r* = 0.984, *p* < 0.01), age and mRNA expressions of *Gfap* (*r* = 0.540, *p* < 0.01), age and Cu storage protein *Mt1a* (*r* = 0.669, *p* < 0.01) and *Mt3* (*r* = 0.806, *p* < 0.01; Table 3). In addition, we also found the following inverse correlations between age and transcriptional expressions of *Nestin* (*r* = −0.584, *p* < 0.01), *Dcx* (*r* = −0.551, *p* < 0.01) and Cu transporter *Ctr1* (*r* = −0.733, *p* < 0.01; Table 3). These age-related correlations indicate that SVZ is capable of accumulating more Cu ions as the age increases.

**Table 3:** **Correlation coefficients of transcriptional expression levels of copper regulatory proteins and cellular markers in SVZ**.

	Age	SVZ-Cu	*Gfap*	*Nestin*	*Dcx*	*Ctr1*	*Dmt1*	*Atp7a*	*Atp7b*	*Cox17*	*Atox1*	*Ccs*	*Mt1a*	*Mt2a*	*Mt3*
Age	1.000	0.984**	0.540**	−0.584**	−0.551**	−0.733**	0.412*	0.231	0.484*	0.308	0.073	0.521*	0.669**	0.365	0.806**
SVZ-Cu		1.000	0.482*	−0.552**	−0.492*	−0.638**	0.422*	0.214	0.556*	0.279	0.042	0.477*	0.712**	0.414*	0.779**
*Gfap*			1.000	−0.622**	−0.999**	−0.701**	−0.037	−0.166	−0.109	0.001	−0.045*	0.120	0.038	−0.225	0.272
*Nestin*				1.000	0.643**	0.585**	−0.071	0.209	−0.160	−0.170	−0.257	−0.174	−0.310	−0.139	−0.438
*Dcx*					1.000	0.715**	0.037	0.164	0.107	0.000	0.037	−0.123	−0.046	0.219	−0.279
*Ctr1*						1.000	0.121	0.131	0.072	0.031	0.062	−0.227	−0.077	0.227	−0.423*
*Dmt1*							1.000	0.731**	0.555**	0.770**	0.558**	0.727**	0.773**	0.773**	0.732**
*Atp7a*								1.000	0.406*	0.564*	0.305	0.592**	0.541*	0.566**	0.452*
*Atp7b*									1.000	0.603**	0.128	0.621**	0.756**	0.772**	0.660**
*Cox17*										1.000	0.523*	0.890**	0.698**	0.772**	0.793**
*Atox1*											1.000	0.257	0.290	0.299	0.352
*Ccs*												1.000	0.726**	0.719**	0.873**
*Mt1a*													1.000	0.905**	0.853**
*Mt2a*														1.000	0.720**
*Mt3*															1.000

*correlation is significant at the p < 0.05 level (2-tailed); **correlation is significant at the p < 0.01 level (2-tailed). n = 6.

**Table 4:** **Correlation coefficients of transcriptional expression levels of copper regulatory proteins in choroid plexus**.

	Age	CP-Cu	*Ctr1*	*Dmt1*	*Atp7a*	*Atp7b*	*Cox17*	*Atox1*	*Ccs*	*Mt1a*	*Mt2a*	*Mt3*
Age	1.000	0.681**	0.632**	0.854**	0.067	−0.157	0.350	−0.576*	0.819**	0.013	−0.058	0.282
CP-Cu		1.000	0.321	0.661**	0.010	−0.164	0.019	−0.403	0.431	0.144	−0.011	0.171
*Ctr1*			1.000	0.743**	0.229	−0.029	0.340	−0.592**	0.631**	−0.265	−0.231	−0.104
*Dmt1*				1.000	0.006	−0.335	0.365	−0.608**	0.814**	−0.149	−0.115	0.063
*Atp7a*					1.000	0.798**	0.320	0.467	0.284	−0.113	0.217	0.421
*Atp7b*						1.000	0.131	0.492*	−0.054	0.030	0.376	0.405
*Cox17*							1.000	0.082	0.727**	−0.132	0.179	0.353
*Atox1*								1.000	−0.301	0.278	0.436	0.440
*Ccs*									1.000	−0.093	0.092	0.410
*Mt1a*										1.000	0.462	0.650**
*Mt2a*											1.000	0.744**
*Mt3*												1.000

*correlation is significant at the p < 0.05 level (2-tailed); **correlation is significant at the p < 0.01 level (2-tailed). n = 6.

When the Cu content in the SVZ was correlated with the mRNA expressions of SVZ cellular markers as well as Cu regulatory proteins, the following positive correlations in the SVZ were identified: Cu content and *Gfap* (*r* = 0.482,*p* < 0.05), Cu content and Cu storage proteins* Mt1a* (*r* = 0.712, *p* < 0.01), *Mt2a* (*r* = 0.414, *p* < 0.05), and *Mt3* (*r* = 0.779, *p* < 0.01; Table 3). The inverse correlations were also observed between: the SVZ Cu content and the expressions of *Nestin* (*r* = −0.552, *p* < 0.01), *Dcx* (*r* = −0.492, *p* < 0.05), and *Ctr1* (*r* = −0.638, *p* < 0.01). The correlation results appear to indicate that Cu regulatory proteins, particularly the Cu storage proteins MTs, are essential for SVZ to concentrate high Cu content. Furthermore, the positive correlation between SVZ Cu content and *Gfap*, and the inverse correlation between SVZ Cu level and *Nestin* as well as *Dcx* suggest that the high Cu environment in the SVZ may affect the neurogenesis within this region.

We also used the same correlation analysis approach to study these correlations in the nearby tissue CP. Results in the Table 4 revealed the following positive correlations between: the age and plexus Cu content (*r* = 0.681, *p* < 0.01), *Ctr1* (*r* = 0.632, *p* < 0.01), *Dmt1* (*r* = 0.854, *p* < 0.01) and *Ccs* (*r* = 0.819, *p* < 0.01). An inverse correlation was observed between the age and *Atox1* (*r* = −0.576, *p* < 0.05). Of the studied Cu transport or storage proteins, only the *Dmt1* expression was found to be positively correlated with plexus Cu content (*r* = 0.661, *p* < 0.01; Table 4). Therefore, the age-related up-regulations of Cu transporters CTR1 and DMT1 and Cu chaperon CCS may contribute to the high Cu accumulation in the CP.

## Discussion

Our current studies confirm our previous observation that the SVZ accumulates remarkably high amounts of Cu as compared with other selected brain regions (Pushkar et al., 2013; Fu et al., 2015). Furthermore, our data demonstrate that Cu contents in all selected brain regions, CSF and serum display an age-related increase. Quantification of the transcriptional expressions of Cu regulatory proteins provides direct evidence of the presence of Cu transporters, chaperons and intracellular binding proteins in the SVZ and CP. Interestingly, we observe that the SVZ is highly enriched with Cu storage MTs, whereas the CP is more abundant with Cu transporters. There are also age-related up-regulation and down-regulations of Cu regulatory proteins in both SVZ and CP. Moreover, significant positive correlations exist between age and Cu contents, and between expression levels of Cu regulatory proteins and Cu content in the SVZ and CP.

Age-related increases of Cu levels in brain, serum and CSF have been reported in literature by studies conducted mainly in rodents and humans (Palm et al., 1990; Zatta et al., 2008; Wang et al., 2010; Hozumi et al., 2011). A recent human study, however, shows an age-related decline in Cu levels in selected brain regions (Ramos et al., 2014). Our current data, along with previous observation (Pushkar et al., 2013), clearly establish an age-related accumulation of Cu in the SVZ, CP and other tested brain regions. With regards to the SVZ and CP, two neighborhood tissues that are separated by the CSF, a high Cu accumulation could be due to: (1) increased Cu uptake; (2) increased intracellular binding or storage of Cu; and/or (3) decreased ouster of intracellular Cu ions. These processes are regulated by a host of proteins whose activities may change with the aging process, which may underscore the age-related buildup of Cu in these tissues. The adjacent anatomic location notwithstanding, the mechanisms by which these two tissues accumulate Cu appear to be entirely different. Our qPCR data revealed an age-dependent increase in expression of Cu storage proteins MTs in the SVZ; whereas these storage proteins showed an age-related reduction in the CP. This difference became even larger in the aged animals; the MTs expressions in the old rats were about 2–14 fold higher in the SVZ than in the plexus. In contrast, the expression of a major Cu uptake transporter Ctr1 showed an age-dependent increase in the CP; but it was greatly reduced in the SVZ. Thus, the different expression patterns between the SVZ and plexus appear to suggest two distinct pathways for Cu to accumulate in respective regions: while the SVZ gains the increased storage ability with aging, the neighborhood CP extends its capability in taking up Cu ions through Cu transporters.

Intracellular storage of Cu is known to be regulated by MTs (Suzuki et al., 2002; Tapia et al., 2004; Ogra et al., 2006). Multiple lines of evidence show that the elevated expression of MTs is the cell’s response to the excess cellular Cu in order to protect the cells against the cytotoxicity induced by redox active Cu ions (Hidalgo et al., 1994; Dincer et al., 1999; Haywood and Vaillant, 2014; Bulcke and Dringen, 2015). It is therefore possible that the observed increase in MTs expression in the SVZ may be a consequence of age-related Cu increase in the body; yet it remains difficult to explain why the MTs expression was reduced in the CP where in fact Cu levels were increased with age.

The CP functions as a barrier between the blood and CSF and transports materials across the BCB. Ctr1 is a major Cu transporter with the highest expression in the apical surface of the choroidal epithelia (Kuo et al., 2006; Zheng and Monnot, 2012; Davies et al., 2013). Our previous work has established that Cu is transported out of brain from the CSF to blood via Ctr1 and DMT1 in the plexus (Zheng and Monnot, 2012). Recent studies by Haywood and Vaillant (2014) on sheep suggest that there is a reverse transfer of Cu from the blood into the choroidal epithelia. The authors further postulate that the elevated Cu content in the aging human brain may be the result of dysregulated CTR1 at brain barriers. However, our own data from adult rats show a distinct apical distribution of Ctr1 in choroidal epithelia (Zheng and Monnot, 2012). It is unclear, though, if this distribution pattern may change over the life time from the apical to the basolateral side of choroidal epithelium. If does, it is quite possible then that the increased expression of Ctr1 in aging rats, as observed in this study, may transport Cu from the blood to the CSF, leading an increased CSF Cu level. Consequently it may act on Cu regulatory machinery in the SVZ. This interesting hypothesis, however, needs further experimentation to prove.

DMT1 in the CP also contributes to cellular Cu uptake, albeit much lesser than Ctr1 in net gain (Zheng et al., 2012). Current data showed that expression of DMT1 appeared to increase significantly with aging in the CP, but to a lesser extent in the SVZ. Literature data have suggested a significant age-related upregulation of DMT1 expression in the FC of 12-month-old APP/PS transgenic mice as compared with the data from 6-month-old animals (Xian-hui et al., 2015). An early study in rat brain also shows an age-dependent alteration in two mRNA isoforms of DMT1, i.e., DMT1 (+IRE) and (−IRE; Ke et al., 2005). Reports by Knutson et al. (2004), however, show no significant age-related change in DMT1 mRNA levels in selected mouse brain regions. While DMT1 may not play an essential role in transporting Cu by the BCB (Zheng et al., 2012), our recent work has indeed exhibited co-localization of DMT1 with the newly proliferating neural stem cells in the SVZ and with neuroblasts in the RMS (Fu et al., 2015). The role of DMT1 in Cu accumulation in the SVZ and related age effect remain to be explored.

Our data raise several interesting questions. First, what cell type in the SVZ is responsible for Cu accumulation? Our correlation analyses revealed a positive correlation between SVZ Cu and *Gfap*, but an inversed relationship with *Nestin* or *Dcx*. GFAP is a cellular marker for glial cells and type B progenitor cells in the SVZ. In the brain, higher Cu contents in glial cells have also been reported by other groups (Szerdahelyi and Kása, 1986; Kodama et al., 1991; Becker and Salber, 2010; Scheiber and Dringen, 2013; Pal and Prasad, 2014). The inverse correlations between SVZ Cu and *Dcx* (marker for type A proliferating neuroblasts) and between SVZ Cu and *Nestin* (marker for type C transit amplifying progenitors), on the other hand, did not support a significant role of either cell type in Cu accumulation in the SVZ. Thus, it seems likely that the age-related increase of Cu in the SVZ occurs primarily in GFAP-positive type B glial cells.

Second, what are the consequences of high Cu levels in the SVZ? It is well known that aging is one of the most relevant factors for the significant decline in adult neurogenesis in the SVZ (Hamilton et al., 2013). The current study has established significant inverse correlations between age and neural stem cell markers of Nestin and Dcx, and between Cu content and both cellular markers. In other words, with increased age, fewer Nestin or Dcx positive-type C or type A cells were proliferated in the SVZ of old rats. These observations support the view of age-related decline in brain’s capability to produce new neurons in neurogenic region—SVZ (Limke and Rao, 2003). Interestingly also, with increased Cu levels, which increases with age, too, fewer type C and type A were proliferated in the SVZ. Thus, these data appear to suggest that extensive Cu accumulation in the SVZ as the function of age may down-regulate the proliferation of neural stem cells, leading to a reduction of neurogenesis.

Finally, what is the exact role of Cu in adult neurogenesis in the SVZ? The data presented in this report have established a regulatory role of Cu in adult neurogenesis. A recent finding by this laboratory has also shown that a significantly reduced Cu content in the SVZ after subchronic Mn exposure in fact enhances the proliferating activity of NSPC in the SVZ and RMS (Fu et al., 2015). Thus, we suspect that in response to changes in biochemical milieu due to aging, environmental exposure or disease states, Cu level in the SVZ may function as a sensor/switch that could either turn on (promote) or turn off (restrain) the signaling pathway that regulates the neurogenesis process in adult brain. This hypothesis requires further studies.

The current study has the following limitations. First, the analyses were based largely on qPCR quantification of translational expression of Cu regulatory proteins. While the established technology provides the confident data, it may not truly reflect amounts of proteins ultimately expressed in tested tissues. Our next step is to use Western blot to determine the protein amounts in the SVZ and CP. In addition, the current study compares the outcomes only between the SVZ and plexus. To fully appreciate Cu’s role in adult neurogenesis, there is a need to compare the distribution of Cu regulatory proteins throughout entire brain including key areas such as HP, STR, and OB etc. Finally, there existed specie differences from rodents to humans. These differences should be taken into account when interpreting the significance of the observed data.

In summary, Cu dyshomeostasis plays an unequivocal role in the pathogenesis of age-related neurodegenerative disorders. The data in this report provides general information on age-dependent Cu content, and expression of various Cu regulatory proteins in the brain, particularly in the neurogenic SVZ and its anatomically adjacent tissue CP. Our observation on increased Cu levels and reduced neurogenesis in the SVZ may provide a new clue to understand the mechanism of adult neurogenesis.

## Conflict of Interest Statement

The authors declare that the research was conducted in the absence of any commercial or financial relationships that could be construed as a potential conflict of interest.
